# Comparison of image quality between a novel mobile CT scanner and current generation stationary CT scanners

**DOI:** 10.1007/s00234-022-03089-3

**Published:** 2022-11-28

**Authors:** Henrik Andersson, Ashkan Tamaddon, Mazdak Malekian, Kristina Ydström, Roger Siemund, Teresa Ullberg, Johan Wasselius

**Affiliations:** 1 Department of Medical Imaging and Physiology, Skåne University Hospital, 221 85 Lund, Sweden; 2 Department of Clinical Sciences, Lund University, 22100 Lund, Sweden; 3 Radiation Physics, Department of Hematology, Oncology and Radiation Physics, Skåne University Hospital, 22185 Lund, Sweden; 4 Medical Radiation Physics Malmö, Department of Translational Medicine, Lund University, 22100 Lund, Sweden

**Keywords:** Mobile CT scanner, Head CT, Point-of-care imaging, Brain CT, Image quality analysis

## Abstract

**Purpose:**

Point-of-care imaging with mobile CT scanners offers several advantages, provided that the image quality is satisfactory. Our aim was to compare image quality of a novel mobile CT to stationary scanners for patients in a neurosurgical intensive care unit (ICU).

**Methods:**

From November 2020 to April 2021, all patients above 18 years of age examined by a mobile CT scanner at a neurosurgical ICU were included if they also had a stationary head CT examination during the same hospitalization. Quantitative image quality parameters included attenuation and noise in six predefined regions of interest, as well as contrast-to-noise ratio between gray and white matter. Subjective image quality was rated on a 4-garde scale, by four radiologists blinded to scanner parameters.

**Results:**

Fifty patients were included in the final study population. Radiation dose and image attenuation values were similar for mobCT and stationary CTs. There was a small statistically significant difference in subjective quality rating between mobCT and stationary CT images. Two radiologists favored the stationary CT images, one was neutral, and one favored mobCT images. For overall image quality, 14% of mobCT images were rated grade 1 (poor image quality) compared to 8% for stationary CT images.

**Conclusion:**

Point-of-care brain CT imaging was successfully performed on clinical neurosurgical ICU patients with small reduction in image quality, predominantly affecting the posterior fossa, compared to high-end stationary CT scanners.

**Supplementary Information:**

The online version contains supplementary material available at 10.1007/s00234-022-03089-3.

## Introduction

Computed tomography (CT) of the head is a cornerstone of diagnosis in neuroradiology due to its high reliability in identifying common pathologies such as ischemia, hemorrhage, and fractures, as well as its high availability and few contraindications [1–4]. CT examinations are predominantly performed using stationary CT scanners, where the patient is brought to the scanner for imaging. For critically ill patients, transportation to a stationary CT can be hazardous, stressful, and time-consuming [5–9].

Mobile CT scanners (mobCT), also referred to as portable CT scanners, are small and maneuverable scanners for point-of-care imaging within the hospital or for prehospital use. The use of mobile scanners has medical, practical, and economic advantages [7, 10–12]. Firstly, point-of-care imaging limits the risk of complications during transportation of patients within the hospital, which for critically ill patients has proven to be a high-risk maneuver with adverse effects occurring in up to 70% of transports [6–9]. Secondly, point-of-care examination has been shown to reduce the time from ordering of the examination to its completion, by as much as two-thirds [12, 13]. Lastly, point-of-care imaging frees up valuable examination time on the stationary CT scanners at the imaging departments [12]. Disadvantages of point-of-care imaging are mainly associated with reduced image quality, which has been demonstrated in previous studies comparing earlier generation mobCT to conventional scanners [14–16].

One area with large potential benefit of point-of-care imaging, and high demands on imaging quality, is neurosurgical intensive care units (neurosurgical ICU). In this setting, frequent evaluation of disease progression is important for determining the correct treatment option and for estimating prognosis. For neurocritical ill patients, close clinical monitoring, including invasive neuromonitoring [17–19], are valuable tools, especially since transportation outside of the neurosurgical ICU may be hazardous or even impossible. However, repeated brain CT imaging remains a crucial and irreplaceable part of the care for many of these patients, and point-of-care imaging in the neurosurgical ICU could fill that need.

The aim of this study was to compare the image quality of a novel mobCT, Somatom On.site (Siemens Healthineers, Erlangen, Germany), with current generation stationary CTs, for a neurosurgical ICU patient population characterized by a high prevalence of intracranial pathology using quantitative and qualitative image analysis.

## Material and methods

### Patient selection

This retrospective single-center study consecutively included all patients undergoing a head CT using a mobCT from its first use in our center on November 9, 2020 to March 31, 2021. Patients under the age of 18 were excluded, as were patients that were not given an additional stationary CT examination during their hospitalization.

For patients undergoing more than one mobCT examination, only the first was included. For patients undergoing more than one stationary CT, the examination closest in time to the mobCT examination was included.

The study was approved by the Swedish Ethical Review Authority (#2021–01,722), and informed consent was waived.

### Image acquisition and reconstruction

The Somatom On.site is a mobile head CT scanner with a 32-row detector, a rotation time of 1.0 s, a tube voltage of 80 or 120 kV, and smallest available section thickness of 0.75 mm. The scanner is transported by a single person and is self-shielding, reducing scatter radiation to nearby staff and patients [20]. The mobCT protocol included iterative reconstruction algorithm SAFIRE grade 4 and reconstruction kernel HR40. The mobCT was a prelaunch version undergoing clinical evaluation, as one of the first three sites in the world.

The stationary CT examinations were performed using one of the following scanners: IQon Spectral CT (Philips healthcare Inc., Best, The Netherlands), Ingenuity and Ingenuity core 128 (Philips Healthcare Inc., Best, The Netherlands), Somatom Definition Flash (Siemens Healthineers, Erlangen, Germany), and Aquilion One Genesis (Canon Medical Systems, Ōtawara, Japan). The protocol settings for all scanners were those currently in clinical use at our center, including vender-specific iterative reconstruction algorithms and reconstruction kernels suitable for imaging of the brain. Table 1 shows the scanner parameters, iterative reconstruction algorithms, and reconstruction kernels used for mobCT and stationary CT examinations.

**Table 1:** CT scanner parameters for the mobCT and the five stationary CTs included in the study. *MobCT* mobile CT

CT scanner parameters	mobCT	Stationary CT				
	Somatom On.site	Somatom Definition Flash	IQon Spectral CT	Ingenuity	Ingenuity Core 128	Aquilion One Genesis
Detector coverage (mm)	24	12	40	40	25	20
Collimation (mm)	32 × 0.75	20 × 0.6	64 × 0.625	64 × 0.625	40 × 0.625	40 × 0.5
Rotation time (s)	1	0.5	0.33	0.4	0.4	0.75
Pitch	0.55	0.55	0.359	0.4	0.4	0.625
Tube voltage (kV)	120	120	120	120	120	120
Tube current	Fixed	ATCM	ATCM	ATCM	ATCM	ATCM
Iterative reconstruction algorithm	Safire 4	Safire 3	IMR2	IMR2	IMR2	AIDR3De
Reconstruction kernel	HR40	J37f	BR	BR	BR	FC26

*mm* millimeters, *s* seconds, *kV* kilovolts, *ATCM* automatic tube current modulation, *Safire 3 and 4* Sinogram Affirmed Iterative Reconstruction levels 3 and 4, *IMR2* Iterative Model Reconstruction level 2, *AIDR3De* Adaptive Iterative Dose Reduction 3D Enhanced, *BR* brain routine, *MobCT* mobile CT

The CT dose index volume (CTDI_vol_) [21, 22] were obtained for all exams.

### Quantitative image analysis

To adhere to the European guidelines [23] and our local practice, image reconstructions were performed with a slice thickness of 4 mm in the axial plane and 3 mm in the coronal and sagittal planes. All image analyses were performed using Sectra IDS7 PACS (Sectra, Linköping, Sweden).

Mean attenuation (HU) and noise levels defined as one standard deviation (1SD) of HU were measured on axial slices in four predefined primary regions of interest (ROIs) representing air, cerebrospinal fluid (CSF), gray matter, and white matter. The air ROI (HU_air_ and 1SD_air_) was placed outside the head at the level of the thalamus and basal ganglia. The CSF ROI (HU_CSF_ and 1SD_CSF_) was placed in the lateral ventricle. The gray matter ROI (HU_GM_ and 1SD_GM_) was placed in the thalamus, and the white matter ROI (HU_WM_PV_ and 1SD_WM_PV_) was placed in the frontal periventricular white mater adjacent to the frontal horn, at the same level of the head as the gray matter ROI. To evaluate if the image noise varied at different levels of the head, two additional white matter ROIs were placed in the centrum semiovale (1SD_WM_CS_) and in the transitional area between the middle cerebellar peduncle and the central white matter of the cerebellum (1SD_WM_C_). The CSF ROI was 5 mm in diameter, since larger ROIs could often not be fitted in the lateral ventricles, while all other ROIs were 10 mm in diameter. All six ROIs were primarily placed on the right side, but in cases of significant pathology or artefacts, a ROI could instead be placed on the right side or in a neighboring area with an equivalent tissue type. Supplemental Fig. 1 illustrates the ROI locations.

The average noise (1SD_avr_) for all ROIs in each CT examination was calculated as the mean of 1SD from all six ROIs. Contrast-to-noise ratio (CNR) between gray and white matter was calculated using HU and 1SD from the primary gray and white matter ROIs, with the white matter being considered the background, which resulted in the following formula being used (HU_GM_-HU_WM_PV)_)/1SD_WM_PV_.

### Qualitative image analysis

All images were rated by four radiologists: two neuroradiology fellows (AT and MM) and two senior neuroradiologists with more than 10 years’ experience (JW and RS).

Four image quality aspects were rated: (1) delineation of gray matter in the basal ganglia, (2) differentiation of gray matter cortex from underlying white matter at the level of the centrum semiovale, (3) differentiation of gray matter cortex from underlying white matter in the cerebellum, and (4) overall image quality.

For each image quality aspect, an ordinal 4-grade text-based scale was used [24]: (1) poor, poor image quality with substantial restriction for clinical use, repeating the examination should be considered; (2) sufficient, sufficient image quality for clinical purposes, although with slight limitations; (3) good, good image quality without significant limitations for clinical use; and (4) excellent, excellent image quality with no limitations for clinical use, better than required for clinical tasks.

Prior to rating the study images, all four raters performed a consensus rating of 20 blinded exams not included in the study dataset, to calibrate themselves with the text-based rating scale. Based on the consensus rating, a combined visual and text-based rating scale was composed with one example image of each grade for three of the four image quality aspects (1–3), which was then made available during the rating of the study images (Supplemental Fig. 2).

All study images were rated independently by each rater on two separate occasions with at least 10 weeks in between, to minimize recall bias. All examinations were manually zoomed and cropped to display similar fields of view and scan lengths before being presented to the raters, who were not allowed to change the zoom. Axial, coronal, and sagittal stacks without scanner parameters, image parameters, or patient information, with a standard window setting (width 80 HU, center 40 HU), were presented in randomized order with an observer noting the grades. The raters were allowed to scroll and adjust the window setting.

### Statistical analysis

IBM SPSS Statistics for Windows version 27 (IBM Corp., Armonk, NY, USA) was used for statistical calculations, and a two-sided *p*-value of < 0.05 was considered significant.

Data was checked for normality using z-test for skewness and kurtosis, as well as visual inspection of Q-Q plots, histograms, and boxplots [25–27]. Paired sample t-tests were performed to compare data that were normally distributed, Wilcoxon signed rank test for not normally distributed data as well as ordinal data, and McNemar change test for nominal data.

Intra-rater agreement, and inter-rater agreement for the first rating session, was assessed for the rating of “Over-all image quality” using quadratic weighted kappa interpreted according to Landis and Koch [28], with values of 0–0.20 as slight, 0.21–0.40 fair, 0.41–0.60 moderate, 0.61–0.80 substantial, and 0.81–1 as almost perfect agreement.

## Results

**Fig. 1:**
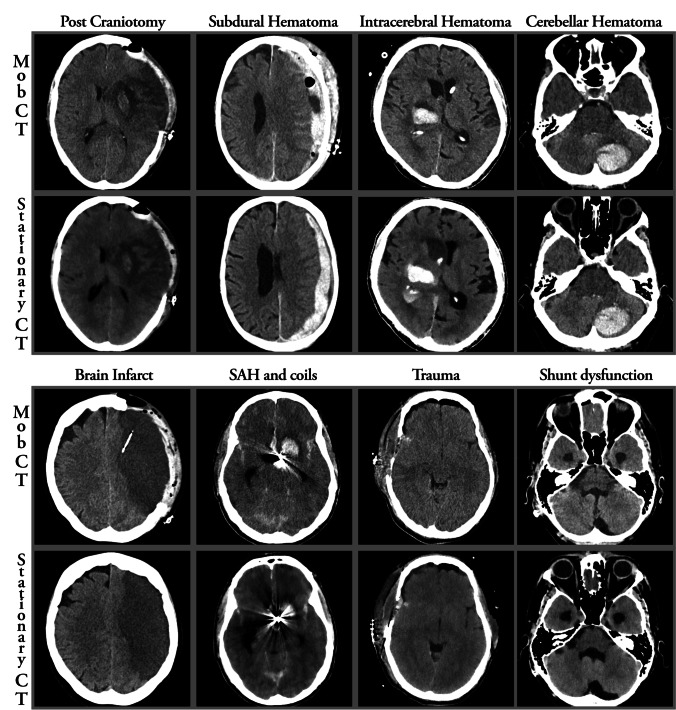
Images chosen to illustrate common findings in mobCT images (upper row) and corresponding stationary CT images (lower row). Upper panel from left to right: post craniotomy, subdural hematoma, intracerebral hematoma, and posterior fossa intracerebral hematoma. Lower panel from left to right: brain infarct, subarachnoid hemorrhage and a coiled aneurysm, trauma (motor vehicle accident), and shunt dysfunction with hydrocephalus

From the first use of the mobCT on November 9, 2021, until March 31, 2021, 61 patients were scanned. Three patients younger than 18 and eight patients who did not undergo an additional stationary CT examination during their hospitalization were excluded. The clinical characteristics of the final study population of 50 patients are shown in Table 2. Figure 1 shows illustrative examples of the findings and of the image quality. The stationary CT scanners included current high-end scanners, with 26 examinations performed on Ingenuity, 10 on Ingenuity Core 128, 6 on Somatom Definition Flash, 6 on IQon Spectral CT, and 2 on Aquilion One. Scanner parameters are shown in Table 1.

**Table 2:** Demographic data of the patients included in the study. Patients could be included in multiple groups of intracranial pathology, depending on the pathology present on the mobCT and/or stationary CT images. *MobCT* mobile CT

Demographic data	Frequency (%) and (1 standard deviation)
Number of patients	50
Female sex	48% (24)
Mean age (SD)	60 years (± 16)
Post neurosurgery	88% (44)
Intracranial hemorrhage	96% (48)
Intracranial foreign bodies	66% (33)
Hydrocephalus	42% (21)
Ischemic infarct	18% (9)
Intra-axial tumors	6% (3)

Quantitative data are presented as *mean* ± *1 SD* if not otherwise stated.

### Radiation dose—CTDI_vol_

MobCT examinations were performed with fixed tube current which gave a constant CTDI_vol_ of 44.1 mGy for all examinations except one that was performed with lower tube current, resulting in a CTDI_vol_ of 24.1 mGy. All stationary CT exams were performed with automatic tube current modulation (ATCM) [29]. There was no statistically significant difference in CTDI_vol_ between mobCT (mean 43.7 ± 2.8) and stationary CT exams (mean 44.0 ± 6.7), *p* = 0.215.

### Quantitative image analysis

#### Attenuation values

Mean attenuation values (HU) for all four measured primary ROIs are shown in Supplementary Table 1. The attenuation of gray matter, white matter, and CSF was slightly higher in mobCT and slightly lower in air. The differences were generally small and in line with the expected values for the examined tissues/substances.

#### Noise level and CNR

**Fig. 2:**
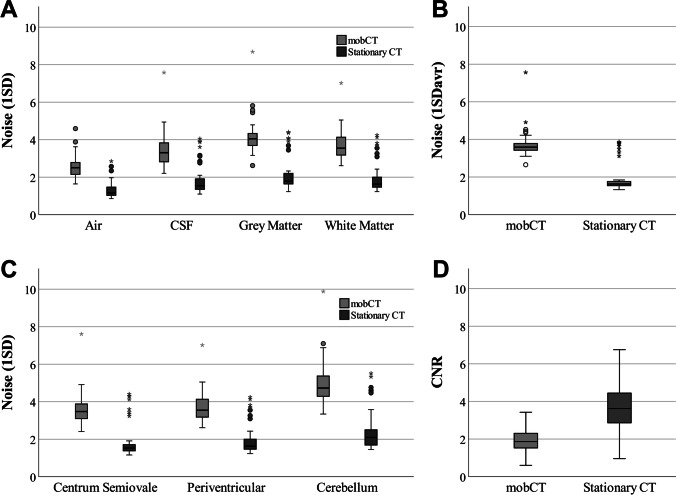
Boxplots comparing images from mobCT and stationary CTs for **a** noise level (1SD) in the four primary regions measured representing air (1SD_air_), CSF (1SD_CFS_), gray matter (1SD_GM_), and white matter (1SD_WM_PV_); **b** average image noise (1SD_avr_) calculated as the average noise in all the six measurement location; **c** noise level (1SD) in white matter measurements at three different levels of the head (1SD_WM_CS_, 1SD_WM_PV_, 1SD_WM_C_); and **d** CNR between gray matter and white matter. The top and bottom box lines represent first and third quartiles, with the middle intersecting line representing the median. The vertical bars represent maximum and minimum values excluding outliers which are shown as circles. Extreme outliers are shown as asterisks. CSF, cerebrospinal fluid; CNR, contras-to-noise-ratio; mobCT, mobile CT

Noise levels for all four primary ROIs (1SD), as well as average noise level (1SD_avr_) and CNR, are shown in Supplementary Table 1 and Fig. 2. Figure 2 additionally shows the noise level (1SD) in the white matter at the three different levels of the head.

For all four primary ROIs, the noise level was significantly higher in mobCT (mean 1SD_air_ 2.5 ± 0.6, mean 1SD_CSF_ 3.4 ± 0.9, mean 1SD_GM_ 4.1 ± 0.9, mean 1SD_WM_PV_ 3.7 ± 0.8) compared to stationary CT images (mean 1SD_air_ 1.3 ± 0.4, mean 1SD_CSF_ 1.8 ± 0.8, mean 1SD_GM_ 2.1 ± 0.9, mean 1SD_WM_PV_ 1.9 ± 0.8), p < 0.001. The noise level parameter 1SD_avr_ was likewise significantly higher in mobCT (mean 1SD_avr_ 3.7 ± 0.7) compared to stationary CT images (mean 1SD_avr_ 1.9 ± 0.8), *p* < 0.001.

When comparing the noise level in white matter at different levels of the brain, the difference was minimal, and not statistically significant, between the measurement in the white matter of the centrum semiovale compared to the periventricular white matter at the level of the basal ganglia, for both mobCT (mean 1SD_WM_CS_ 3.6 ± 0.8 and mean 1SD_WM_PV_ 3.7 ± 0.8) and stationary CT (mean 1SD_WM_CS_ 1.9 ± 0.9 and mean 1SD_WM_PV_ 1.9 ± 0.9), *p* = 0.318 and *p* = 0.053, respectively. However, the difference was larger, and statistically significant, when comparing the noise at both levels to the noise in the white matter in the posterior fossa for mobCT (mean 1SD_WM_C_ 5.0 ± 1.1) and stationary CT images (mean 1SD_WM_C_ 2.4 ± 1.0), *p* < 0.001. When comparing the absolute difference in noise between the white matter of the centrum semiovale and the white matter of the posterior fossa for each type of CT, the difference was significantly larger for mobCT (mean difference 1.4 ± 0.8) compared to stationary CT (mean difference 0.6 ± 0.4), *p* < 0.001.

Lastly, the CNR was significantly lower in mobCT images (mean CNR 1.9 ± 0.6) compared to stationary CT images (mean CNR 3.6 ± 1.2), *p* < 0.001.

### Qualitative image analysis

#### Intra- and inter-rater agreement

Intra- and inter-rater agreement is shown in Supplementary Table 2. The intra-rater agreement was *substantial* for radiologist 1 (0.68) and *moderate* for radiologist 2, 3, and 4 (0.51, 0.55, and 0.54). The inter-rater agreement between all six possible pairs of radiologists ranged from *slight* to *moderate* (0.19–0.59), indicating diverging opinions.

#### Subjective image quality rating

**Fig. 3:**
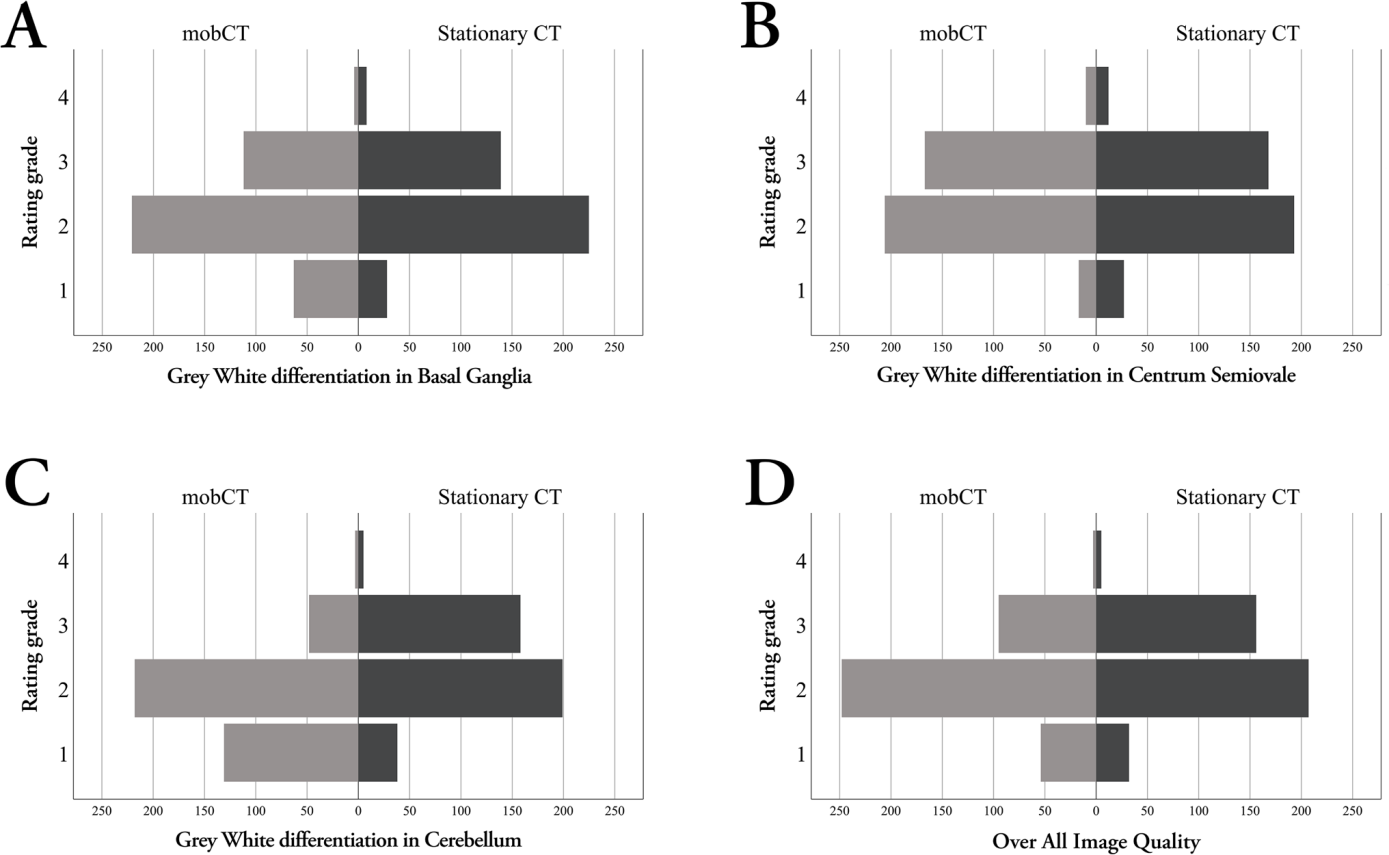
Histogram showing rating frequency for all four raters combined, comparing mobCT to stationary CT images for **a** delineation of gray matter in the basal ganglia, **b** differentiation of gray matter cortex from underlying white matter at the level of the centrum semiovale, **c** differentiation of gray matter cortex from underlying white matter in the cerebellum, and **d** overall image quality. MobCT, mobile CT

All 100 CT examinations were rated by all four radiologists on two separate occasions, with 83 to 117 days between the rating sessions. The count, frequencies, and mean of rating grades for all four image quality aspects are listed in Table 3, and the distribution is shown in Fig. 3. For all radiologists and all four image quality aspects combined, there was a small but statistically significant difference in rating between mobCT (mean rating grade 2.1) and stationary CT (mean rating grade 2.4), *p* < 0.001.

**Table 3:** Mean rating grade of image quality from mobCT and stationary CTs. Shown are results for all raters combined, divided per image quality aspect rated, as well as total of all image quality aspects. Numbers within parentheses represent percent of total rating count for each CT type and image quality aspect. *MobCT* mobile CT

Grade	Image quality aspect									
	1. Gray/white discrimination in basal ganglia		2. Gray/white discrimination in centrum semiovale		3. Gray/white discrimination in cerebellum		4. Overall image quality		Total	
	mobCT	Stationary CT	mobCT	Stationary CT	mobCT	Stationary CT	mobCT	Stationary CT	mobCT	Stationary CT
1	63 (16)	28 (7)	17 (4)	27 (7)	131 (33)	38 (10)	54 (14)	32 (8)	265 (17)	125 (8)
2	221 (55)	225 (56)	206 (52)	193 (48)	218 (55)	199 (50)	248 (62)	207 (52)	893 (56)	824 (52)
3	112 (28)	139 (35)	167 (42)	168 (42)	48 (12)	158 (40)	95 (24)	156 (39)	422 (26)	621 (39)
4	4 (1)	8 (2)	10 (3)	12 (3)	3 (1)	5 (1)	3 (1)	5 (1)	20 (1)	30 (2)
Mean	2.1	2.3	2.4	2.4	1.8	2.3	2.1	2.3	2.1	2.4

For the first image quality aspect “Delineation of gray matter in the basal ganglia,” there was a small statistically significant difference between mobCT (mean rating grade 2.1) and stationary CT (mean rating grade 2.3), *p* < 0.001.

For the second image quality aspect “Differentiation of gray matter cortex from underlying white matter at the level of the centrum semiovale,” there was no significant difference between mobCT (mean rating grade 2.4) and stationary CT (mean rating grade 2.4), *p* = 0.74.

The third image quality aspect “Differentiation of gray matter cortex from underlying white matter in the cerebellum” showed the largest difference in rating grade between mobCT (mean rating grade 1.8) and stationary CT (mean rating grade 2.3), *p* < 0.001.

For the fourth image quality aspect “Overall image quality,” there was also a small significant difference between mobCT (mean rating grade 2.1) and stationary CT (mean rating grade 2.3), *p* < 0.001.

The images obtained by mobCT received a total of 265 grade 1 ratings (17%) compared to only 125 (8%) obtained by stationary CT, which was a statistically significant difference, *p* < 0.001. However, the distribution of grade 1 ratings was very different for mobCT compared to the stationary CT. For mobCT images, 131 (49%) of the grade 1 ratings were given on image quality aspect 3 “Differentiation of gray matter cortex from underlying white matter in the cerebellum” compared to 38 (30%) for the stationary CT images. For the other image quality aspects, the difference was small, with mobCT images receiving fewer grade 1 ratings compared to stationary CTs for image quality aspect 2 “Differentiation of gray matter cortex from underlying white matter at the level of the centrum semiovale,” as shown in Table 3.

There was considerable variation between the four raters. Two of the radiologists, raters 1 and 4, rated image quality significantly lower for mobCT (mean rating grades 1.9 and 2.0, respectively) compared to stationary CT (mean rating grades 2.4 and 2.6, respectively), *p* < 0.001. Rater 2 rated the image quality of mobCT (mean rating grade 2.2) marginally higher than stationary CT (mean rating grade 2.1), but the difference was not statistically significant, *p* = 0.296. Contrary to raters 1 and 4, rater 3 rated the image quality of mobCT (mean rating grade 2.5) significantly higher than stationary CT (mean rating grade 2.3), *p* < 0.001.

## Discussion

The aim of this study was to compare image quality of a novel mobCT with current generation stationary CTs for a neurosurgical ICU patient population characterized by high prevalence of intracranial pathology, using quantitative and qualitative image analysis.

There was no significant difference in radiation dose, which otherwise might have influenced the image quality [21, 22]. The mean attenuation for air, CSF, gray matter, and white matter were similar between mobCT and stationary CT, with high consistency throughout the examinations in the study indicated by a low standard deviation for each value. As expected, the image noise level was significantly higher in mobCT images for all four primary ROIs, indicating higher noise levels throughout the density spectra from air to gray matter. When comparing the noise at different levels of the brain, both mobCT and stationary CT images showed a statistically significant increase in noise in the posterior fossa compared to at the two more superior levels. In our experience, it is common for CT scanners to suffer from reduced image quality at the level of the posterior fossa, compared to more superior levels of the brain. This is likely in part due to surrounding high-density structures, such as the petrous part of the temporal bones, giving rise to beam hardening artefacts and potentially a reduction in the total number of photons reaching the detectors. As a direct result of the higher noise, the mean CNR between gray and white matter was 47% lower for mobCT images, which may potentially reduce the detection of low contrast lesions, such as early signs of ischemia.

For the qualitative image analysis, two senior neuroradiologists and two neuroradiology fellows were included to represent different levels of experience. The intra-rater agreement was *moderate* to *substantial* for the four raters, likely aided by the preparatory consensus rating session and the use of a combined visual and text-based rating scale with image examples. The inter-rater agreement was *slight* to *moderate* for the six possible combinations of rater pairs, where two raters (one fellow and one senior neuroradiologist) favored stationary CT images, one rater favored mobCT images, and one rater was neutral. One contributing reason to the raters’ diverging opinions could be a personal preference for specific vendors’ image characteristics, since the mobCT images were from a single CT scanner and vendor, whereas the stationary CT images were from multiple scanners and vendors. This is an inherent challenge when comparing images from different CT models or vendors; however, it is our belief that the rating results of all four raters combined accurately depict the qualitative image quality difference between the two types of scanners, as specified below.

The combined subjective rating of all raters showed a small but statistically significant difference, with the mean rating for “Overall image quality” being significantly lower for mobCT than for stationary CT images. However, there were substantial differences among the image quality aspects rated, ranging from no difference in “Differentiation of gray matter cortex from underlying white matter at the level of the centrum semiovale” to a significant but small difference in “Delineation of gray matter in the basal ganglia” and a significant and larger difference for “Differentiation of gray matter cortex from underlying white matter in the cerebellum.” This is compatible with the results of the quantitative image analysis, where mobCT showed a significantly larger increase in noise between the white matter of the centrum semiovale and the white matter in the posterior fossa, compared to stationary CT. One potential explanation may be that the stationary CTs used ATCM, which automatically adjusts the tube current based on the thickness of the head to maintain an even predefined image quality [29], while the mobCT uses a fixed tube current regardless of the thickness of the head. Considering that the CTDI_vol_ was similar in the mobCT and stationary CT images, the mobCT radiation level would be relatively higher at the convexities, where the diameter of the head is the smallest, and relatively lower at the thicker parts of the head, such as at the level of the basal ganglia and the posterior fossa. This could explain why mobCT cortical gray-white matter differentiation was equal to that of the stationary CT at the level of the centrum semiovale but inferior in the basal ganglia and markedly inferior in the cerebellum.

When comparing the total number of grade 1 ratings for mobCT versus stationary CT images, the difference was statistically significant. Furthermore, where grade 1 ratings were evenly distributed between all image quality aspects for the stationary CT, the distribution was markedly uneven for mobCT, with almost half of grade 1 ratings given for “Differentiation of gray matter cortex from underlying white matter in the cerebellum.” This again suggests that the posterior fossa is a region of concern for this mobCT, which should be kept in mind when assessing the images.

When reviewing the subjective image quality rating in this study, it is also worth noting that the ratings were fairly low overall, with a mean grade of 2.1 for the mobCT images and 2.4 for the stationary CT images. One reason for this could be the high prevalence of severe intracranial pathology which distorts the normal anatomy and may reduce the overall perception of image quality. Furthermore, in neurosurgical ICU patients, extracranial and intracranial lines, tubes, and clips often give rise to artefacts, which also may reduce the subjective perception of image quality. Another potential reason may be that the consensus reading resulted in too high of a baseline, with raters having higher expectations of image quality compared to when reading images clinically.

The results of this study are in line with the results of previous studies comparing earlier types of mobCT to then contemporary stationary CTs. Matson et al. found the image quality of the mobCT Tomoscan M (Philips healthcare Inc., Best, The Netherlands), first launched in 1995, to be comparable to stationary CTs, although with higher noise [15]. However, when reviewing the example images, it is apparent that the image quality of this earlier generation mobCT is vastly inferior to modern CTs, why the comparison might not be fully relevant. Abdullah et al. and Rumboldt et al. evaluated CereTom (NeuroLogica, Danvers, MA, USA), which was first launched in 2005, and found the image quality to be inferior to stationary CTs but still acceptable for clinical use. Similar to our study, the presented results indicated an inferior image quality at the level of the middle cerebellar peduncles, with lower gray matter to white matter differentiation as well as more streak artefacts and noise, compared to at the level of the centrum semiovale and the basal ganglia [14, 16]. The underlying reasons for this were, however, not further discussed in the articles.

Limitations of this study include being a single-center study where local factors such as scanner protocols, reconstructions, and PACS used might not be fully generalizable to other centers. Another limitation is that our study analyzed image quality based on mean HU, noise levels, and CNR as well as subjective rating of image quality, largely based on normal anatomical structures. It is our belief that this type of analysis translates well into prerequisites for lesion detection, but further studies should also include diagnostic accuracy for specific pathological findings such as detection of ischemia or subarachnoid hemorrhage to confirm this. All patients included in the study were critically ill and needing neurosurgical ICU treatment, with a very high prevalence of intracranial pathology. Therefore, the results may not be fully generalizable to other patient populations where a lower frequency of intracranial pathology is expected. Furthermore, since the mobCT and stationary CT examinations were not performed simultaneously for each patient, intracranial status may have changed in between the two exams. Lastly, another limitation is that the study did not analyze transportation, preparation, examination times, or other factors that are part of the rationale for point-of-care imaging using mobCT scanners.

## Conclusion

In this study, point-of-care brain imaging was successfully performed on clinical neurosurgical ICU patients using a mobCT. Image analysis showed a small reduction in image quality, predominantly affecting the posterior fossa, compared to high-end stationary CT scanners.

## Supplementary Information

ESM 2 (PDF 1.81 MB)

## Data Availability

An aggregatet dataset without personal information man be provided upon reasonable request including relevant Ethical approval.
