# Supplementary material for: Comparison of image quality between a novel mobile CT scanner and current generation stationary CT scanners
Source: Neuroradiology. 2022 Nov 28;65(3):503–12. doi: 10.1007/s00234-022-03089-3 (PMC9905188; doi:10.1007/s00234-022-03089-3)
Supplement: Supplementary file 1 — (PDF 1.81 MB) [file 234_2022_3089_MOESM1_ESM.pdf]

Supplementary Table 1

Region	Air		CSF		Grey Matter		White Matter		Combined	
**CT Type**	mobCT	Stationary CT	mobCT	Stationary CT	mobCT	Stationary CT	mobCT	Stationary CT	mobCT	Stationary CT
**HU**									**CNR**	
Mean	-1006	-1001	10	5	38	33	31	27	1.9	3.6
SD	3	3	2	2	2	2	2	2	0.6	1.2
Minimum	-1023	-1005	3	1	34	28	23	19	0.6	1.0
Maximum	-1000	-980	13	12	41	39	35	33	3.4	6.8
Range	23	25	10	11	7	11	12	14	2.8	5.8
**1SD**									**$1SD_{avr}$**	
Mean	2.5	1.3	3.4	1.8	4.1	2.1	3.7	1.9	3.7	1.9
SD	0.6	0.4	0.9	0.8	0.9	0.9	0.8	0.8	0.7	0.8
Minimum	1.6	0.9	2.2	1.1	2.6	1.2	2.6	1.2	2.7	1.3
Maximum	4.6	2.9	7.6	4.1	8.7	4.4	7.0	4.2	7.6	3.9
Range	3.0	2.0	5.4	3.0	6.1	3.2	4.4	3.0	4.9	2.5

CNR = contrast-to-noise-ration between grey matter and white matter, CSF = cerebrospinal fluid, HU =Attenuation value in Hounsfield Units, 1SD = Noise level in Hounsfield Units, $1SD_{avr}$ = Average noise level in images in Hounsfield Units, SD = Standard deviation

Supplementary Table 1.

Attenuation values and noise level for mobCT and stationary CTs for each of the four primary regions of interest measured representing air (HU_{air} and $1SD_{air}$), CSF (HU_{CFS} and $1SD_{CFS}$), grey matter (HU_{GM} and $1SD_{GM}$) and white matter (HU_{WM_PV} and $1SD_{WM\_PV}$), CNR between grey matter and white matter, and average image noise level ($1SD_{avr}$). MobCT = mobile CT.

Supplementary Table 2

Intra-rater Agreement	Quadratic Weighted Kappa	95% Asymptotic Confidence Interval	
		Lower Bound	Upper Bound
Rater 1, session 1 vs 2	0,68	0,56	0,80
Rater 2, session 1 vs 2	0,51	0,34	0,67
Rater 3, session 1 vs 2	0,55	0,43	0,67
Rater 4, session 1 vs 2	0,54	0,41	0,67
**Inter-rater Agreement Session 1**	**Quadratic Weighted Kappa**	**95% Asymptotic Confidence Interval**	
		**Lower Bound**	**Upper Bound**
Rater 1 vs Rater 2	0,30	0,12	0,47
Rater 1 vs Rater 3	0,26	0,07	0,44
Rater 1 vs Rater 4	0,59	0,47	0,71
Rater 2 vs Rater 3	0,56	0,46	0,67
Rater 2 vs Rater 4	0,24	0,08	0,39
Rater 3 vs Rater 4	0,19	0,05	0,37

Supplementary Table 2.

Intra-and inter-rater agreement for each radiologist assessing “Overall image quality” of mobCT and stationary CT images. Agreement was assessed using Cohen´s Weighted Kappa with quadratic weights. Intra-rater agreement was calculated for each radiologist between rating session 1 and rating session 2. Inter-rater agreement was calculated between each of the six possible rater pairs for rating session 1. MobCT = mobile CT. Interpretation according to Landis and Koch (23); values of 0-0.20 slight, 0.21-0.40 fair, 0.41-0.60 moderate, 0.61- 0.80 substantial, and 0.81-1 as almost perfect agreement.

Supplementary Figure 1

ROI placement examples

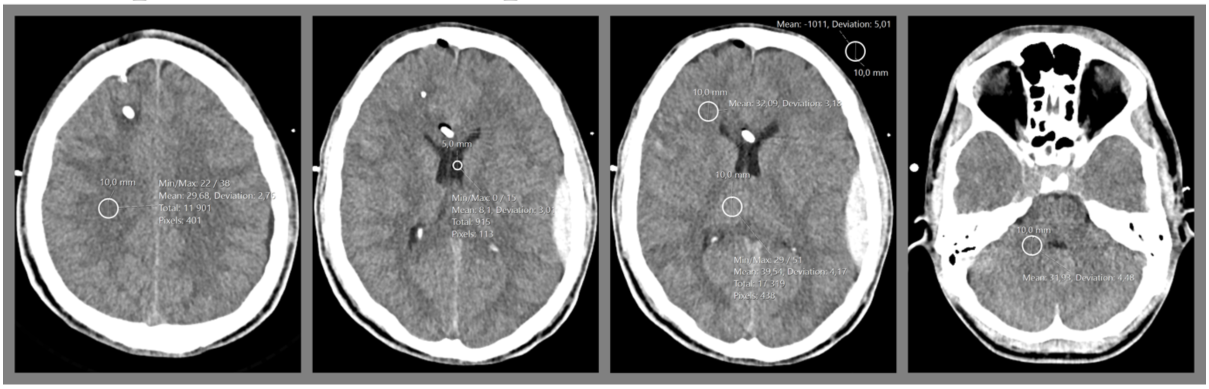

Supplemental Figure 1.

ROI placements.

Supplementary Figure 2

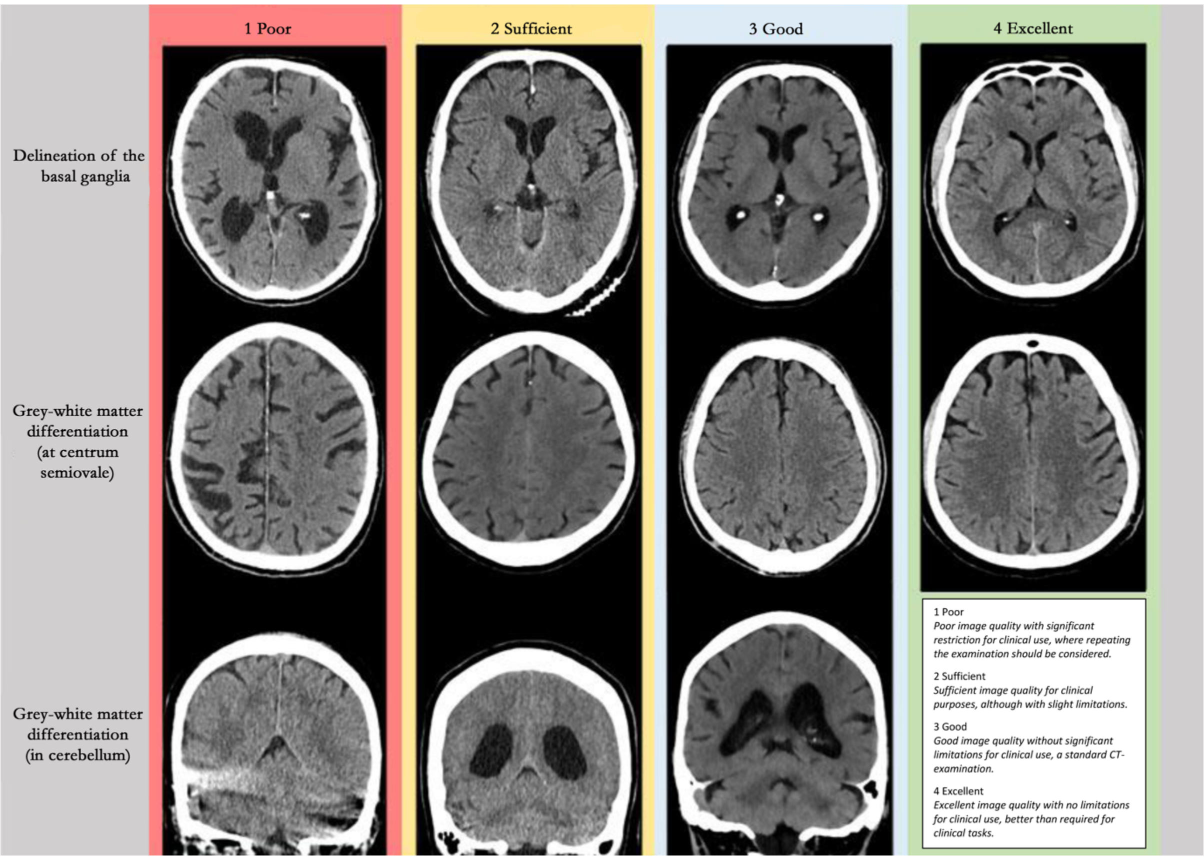

Supplementary Figure 2.

Combined image and text-based grading scale displayed to the raters while rating the study images. All image examples are derived from the consensus rating performed by all four raters prior to rating the study population. Rows represent three out of the four image quality aspects rated. Image quality aspect 4 “Over all image quality” is excluded, since one image cannot justifiably represent this more generalized assessment. Columns represents the four rating grades. In the bottom right corner is the text-based rating scale.
